# Update on Obesity in Psoriasis Patients

**DOI:** 10.3390/life13101947

**Published:** 2023-09-22

**Authors:** Dan Vata, Bogdan Marian Tarcau, Ioana Adriana Popescu, Ioana Alina Halip, Adriana Ionela Patrascu, Dragos-Florin Gheuca Solovastru, Madalina Mocanu, Petronela Cristina Chiriac, Laura Gheuca Solovastru

**Affiliations:** 1 Department of Dermatology, “Grigore T. Popa” University of Medicine and Pharmacy, 700115 Iasi, Romania; dan.vata@umfiasi.ro (D.V.); ioana-alina.grajdeanu@umfiasi.ro (I.A.H.); adriana.patrascu@umfiasi.ro (A.I.P.); madalina.mocanu@umfiasi.ro (M.M.); solovastru.gheuca@umfiasi.ro (L.G.S.); 2 Dermatology Clinic, “St. Spiridon” County Emergency Clinical Hospital, 700111 Iasi, Romania; 3 “Grigore T. Popa” University of Medicine and Pharmacy, 700115 Iasi, Romania; 4 “St. Spiridon” County Emergency Clinical Hospital, 700111 Iasi, Romania; cristinachiriac73@gmail.com

**Keywords:** psoriasis, obesity, adipokines, autoimmune, microbiome

## Abstract

Psoriasis is a chronic inflammatory skin condition, with genetic, epigenetic, environmental, and lifestyle factors contributing to its onset and recurrence. Severe psoriasis has a great impact on quality of life, which is similar to that of insulin-dependent diabetes, depression, and ischemic heart disease, but with a lower mortality. There is an overlap between the rising incidences of autoimmune diseases and obesity. In recent years, research has shown that there is an association between psoriasis and obesity. Psoriasis is linked to obesity in a two-way manner, as each can precipitate the development of the other. Several adipose tissue-secreted adipokines were shown to be elevated in obese psoriasis patients, exhibiting similar mechanisms of action to those underlying the pathogenesis of psoriasis. Excess body weight can influence not only the treatment response in psoriasis, but also the adverse events, leading to decreased patient compliance. Specific human microbiome patterns have been identified for obesity and psoriasis and could represent a future therapeutic target in selected individuals.

## 1. Introduction

Psoriasis is a chronic inflammatory skin condition, with a worldwide prevalence varying from 0.91% to 8.5% [1]. The onset and recurrences of psoriasis can be attributed to genetic, epigenetic, environmental, and lifestyle factors [2]. The immune response in psoriasis is characterized by proliferation of Th1, Th17, and Th22 cells resulting in the production of the pro-inflammatory mediators interferon-γ, tumor necrosis factor (TNF)-α, interleukin (IL)-6, and IL-22 [3].

Given the systemic inflammatory status of the psoriasis patients, several comorbidities, such as cardiovascular diseases, metabolic syndrome, obesity, diabetes mellitus, and psoriatic arthritis, are associated [4,5].

From a clinical point of view, patients with psoriasis have erythematous, scaly patches, and plaques localized at the level of elbows, knees, on the scalp, and lower back, but any part of the body may be affected. Severe psoriasis exerts a significant influence on one’s quality of life, comparable to the impact of conditions such as insulin-dependent diabetes, depression, and ischemic heart disease. However, the mortality associated with severe psoriasis is comparatively lower than that of these conditions.

Psoriasis remains an incurable condition, where the physical burden is compounded by the psychological challenge. Stigma, social anxiety (43%), negative self-image, depression (62%), and daily physical function limitations (43%) are observed in psoriasis patients [6,7,8]. These symptoms are present especially when the lesions are distributed on more visible or sensitive areas such as the arms, hands, nails, genitals, head, and neck [6,9]. Moreover, lesions located on these areas are frequently subject to inaccurate diagnosis and insufficient treatment [10].

Autoimmune diseases have presented an increasing trend in prevalence in the last decades among industrialized countries [11]. The same trend has been observed regarding obesity; hence, the link between the two health conditions has become increasingly more obvious [12]. 

Obesity is an important health problem in which excess body fat accumulates, and this can have a negative impact on overall health. The diet trend in industrialized countries promotes high-fat, high-salt, high-sugar diets with excess caloric intake, resulting in an obesity epidemic. Obesity can be diagnosed by calculating the body mass index (BMI), which is the weight in kilograms divided by the square of the height in meters. The World Health Organization classifies BMI in adults as follows: a BMI between 18.5 and 24.9 is normal, 25–29 indicates overweight, and a BMI of more than 30 denotes obesity [13]. In the fight against obesity, nutritional education and adopting an active lifestyle play crucial roles. Promoting a balanced diet, regular exercise, and health awareness can help prevent and manage obesity, while improving quality of life and promoting overall well-being.

New evidence has emerged that the obese phenotype should not be based on only the BMI value, but rather on the metabolic state of the individual, as a metabolically unhealthy normal weight phenotype has been recognized and is at risk for meta-inflammation [14,15]. 

The white adipose tissue secretes several types of adipokines, which are involved in metabolism, appetite regulation, inflammation, and immunity [16]. The most studied adipokines in psoriasis are adiponectin, leptin, and resistin [17]. Due to the pro-inflammatory action of the adipokines (except adiponectin, which is anti-inflammatory), obesity is considered a low-grade inflammatory disease, which represents the foundation of several comorbidities [18].

The aim of this PubMed-based review is to provide an up-to-date evaluation of the association between psoriasis and obesity and the influence of obesity on psoriasis treatment.

## 2. Materials and Methods

This narrative review on the link between psoriasis and obesity was based on articles retrieved from PubMed, Scopus, EMBASE, ISI Web of Science, ScienceDirect, and Cochrane Library Plus databases up until August 2023. All articles used were in English. The main keywords used for the research were “psoriasis”, “psoriasis and obesity”, “psoriasis and adipokines”, “autoimmune”, and “psoriasis microbiome”. Inclusion criteria were as follows: text in English and full text available. Exclusion criteria were as follows: other languages than English and missing full text. In the first phase, the abstracts of the articles were reviewed, and those not relevant to the subject were excluded. In the second phase, a total of 107 articles were reviewed in full text.

## 3. Psoriasis and Obesity

Overweight and especially obesity are recognized as independent risk factors for psoriasis development, but psoriasis itself could precipitate obesity [19]. An overview of the pathophysiology of psoriasis and obesity is presented in Table 1.

Psoriasis and obesity are two distinct yet interconnected conditions that share complex pathophysiological mechanisms, often forming a vicious cycle that exacerbates each other’s effects. Understanding the intricate interplay between these conditions is crucial for effective management and treatment.

Psoriasis is a chronic autoimmune skin disorder characterized by an increased epidermal turnover, resulting in thick, erythematous, scaly patches. 

Obesity, on the other hand, is a metabolic disorder characterized by excess adipose tissue accumulation. The pathophysiology of obesity involves a complex interplay of genetic, environmental, and hormonal factors. 

The connection between psoriasis and obesity lies in the shared inflammatory pathways. Adipose tissue inflammation in obesity exacerbates the immune dysregulation seen in psoriasis, leading to an increased risk of psoriasis development and exacerbation [17,24,25]. Conversely, psoriatic inflammation can lead to insulin resistance and metabolic dysfunction, promoting weight gain and obesity [26]. Additionally, systemic treatments for psoriasis, such as corticosteroids, may contribute to weight gain, further complicating the relationship between the two conditions.

**Figure 1:**
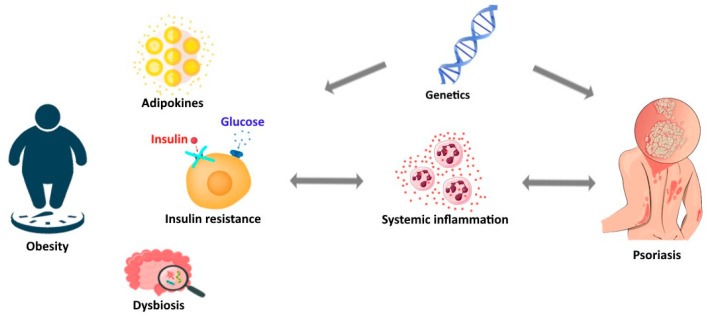
Schematic representation of the bidirectional link between psoriasis and obesity.

Figure 1 displays a schematic representation of the bidirectional link between psoriasis and obesity.

A meta-analysis that included 201,831 psoriasis patients reported an odds ratio (OR) of 1.66 (95% confidence interval 1.46–1.89) for obesity among these patients, in comparison to those without psoriasis [27]. In addition to body mass index (BMI), other obesity measurement tools such as waist circumference (WC), waist-to-hip ratio, and weight gain were associated with a higher risk of developing psoriasis [28]. The relative risk was 1.19 for a 5-unit increase in BMI, 1.24 for a 10-cm increase in WC, 1.37 for a 0.1-unit increase in waist-to-hip ratio, and 1.11 for a 5-kg increase in weight [29].

Langan et al. reported a “dose-dependent” relationship between obesity and the severity of psoriasis, based on the body surface area involved. In comparison with the controls, the patients with mild, moderate, and severe psoriasis had an obesity prevalence increased by 14%, 34%, and 66%, respectively [30].

In a Korean prospective cohort study on 399,461 psoriasis patients, those with a BMI >30 posed a higher risk of psoriasis (hazards ratio 1.118) than those with normal weight. Subjects with a WC of over 105 cm were associated with the highest risk of psoriasis and the male patients with a high WC (90+), while subjects with normal BMI had a greater risk of psoriasis than those with a high BMI [31]. The authors suggested that WC, as a marker of abdominal obesity, had a better positive predictive value of incident psoriasis than BMI [31]. The values used for defining a high WC in the Korean population are lower than the standard European ones (men 90 vs. 102 cm and women 85 vs. 88 cm). 

A cross-sectional study on the prevalence of psoriasis among 887,765 Israeli adolescents reported an increased OR of 1.34 and 1.56 for the overweight and obese adolescents, respectively; a lower OR of 0.8 was associated with a BMI < 20 [32].

Koebnick et al. studied the ORs for psoriasis in different children weight categories: 0.68—underweight, 1.00—normal weight, 1.31—overweight, 1.39—moderately obese, 1.78—extremely obese [33]. In the same study, after the adjustment for BMI, it was found that mean total cholesterol, low-density lipoprotein cholesterol, triglycerides, and alanine aminotransferase had significantly higher values in the psoriasis subjects than in those without psoriasis [33].

A normal BMI associated with central adiposity was also found to be a risk factor for children developing psoriasis, with an OR of 2.77 (23.0% vs. 9.7% in controls) [34].

According to the latest study on the prevalence of psoriasis in the general population of Romania (4.99%; N = 1500), psoriasis was not significantly associated with obesity [35]. These results may be due to the stigma from which obese patients suffer, who are reluctant to come for an office visit.

Obesity may play a significant role in the progression from skin psoriasis to psoriatic arthritis (PsA). Multiple studies propose that obesity serves as a common risk factor for the development of both psoriasis and PsA. In a cohort study utilizing an electronic medical records database that accurately represents the broader UK population, spanning a 15-year period, it was observed that the occurrence rates of PsA rose in correlation with BMI. This trend was evident not only within the group of 75,395 individuals with psoriasis but also within the nearly 2 million individuals from the general population [36]. In the study conducted by Li et al. [37], data concerning body mass index (BMI), fluctuations in weight, and indicators of central obesity were examined in participants of the US Nurse Health Study II, involving 89,049 women over a span of 14 years. The findings revealed a consistent link between higher BMI and an elevated likelihood of developing PsA. Furthermore, a progressively positive correlation was established between changes in weight starting from the age of 18 and markers of central obesity, accentuating the risk of PsA. A similar connection was identified among participants who developed psoriasis during the study’s follow-up period.

## 4. The Molecular Link between Psoriasis and Obesity

Adipokines can be roughly categorized into two main groups: those that are considered “metabolically unfavorable”, such as resistin, and those that are deemed “metabolically advantageous”, including leptin and adiponectin [38].

Leptin, an adipokine with appetite control, pro-inflammatory, and atherogenic properties, was found to be increased in obese patients. Because leptin serves as an indicator of stored energy levels, it acts to suppress food intake. However, when there is leptin resistance (where high levels of leptin do not operate effectively), it can result in unregulated eating and subsequent weight gain, further exacerbating insulin resistance [38]. An increased production of T helper 1 and IL-17A cytokines, which play a central role in the pathogenesis of psoriasis, was associated with leptin [24]. Several studies on psoriasis patients reported high levels of leptin, which correlated with obesity and the severity of the disease [39]. Kyriakou et al. reported no changes in leptin levels following systemic or topical therapy [40].

An analysis of over 3000 psoriasis patients concluded that adiponectin, an anti-inflammatory adipokine, presented decreased levels in the psoriasis patients with concomitant overweight/obesity, diabetes, or metabolic syndrome [41]. Adiponectin plays a protective role in preventing insulin resistance [42]. It operates by suppressing inflammation and has demonstrated the capability to reduce the synthesis of TNF-α and IL-6, while concurrently boosting the generation of anti-inflammatory agents such as IL-10 [43]. Research has indicated an inverse relationship between adiponectin and TNF-α [44]. TNF-α is recognized for its ability to hinder the generation of adiponectin [44], which explains the lower levels of adiponectin observed in individuals with psoriasis compared to healthy individuals. The adiponectin levels did not correlate with the severity of psoriasis and remained stable over the 52 weeks of treatment with secukinumab or etanercept [41]. 

Resistin is a pro-inflammatory and atherogenic adipokine; however, unlike leptin and adiponectin, it can also be produced by keratinocytes. Resistin was shown to correlate with the severity of psoriasis and to enhance the production of tumor necrosis factor-α (TNF- α) and interleukin-6 (IL-6), both of them being part of the pathogenesis of psoriasis [17]. Coban et al. found an association between BMI and serum levels of resistin [45]. This finding is inconsistent among researchers, some of whom also positively correlated resistin with the WC [46]. Phototherapy and systemic treatments, including methotrexate (MTX), acitretin, adalimumab, etanercept, infliximab, and ustekinumab, were proven to reduce the levels of resistin, which is of clinically significant importance [40]. This result could be a consequence of the improved keratinocyte secretion of resistin, without any therapeutic adipocyte functional changes. In addition to psoriasis treatment monitoring by imaging techniques, as well as C-reactive protein, erythrocyte sedimentation rate, fibrinogen, zinc, and copper levels, resistin could also be a valuable predictive marker [47,48,49].

The adipose tissue is also responsible for the direct secretion of IL-1β, IL-6, TNF-α and monocyte chemoattractant protein-1 [25]. It is important to note that these pro-inflammatory cytokines have the capacity to both promote and suppress the production of “harmful” and “favorable” adipokines, respectively [38].

## 5. The Impact of Obesity on the Psoriasis Treatment

The connection between psoriasis and obesity goes beyond their coexistence, as obesity has been found to significantly impact the effectiveness of psoriasis treatment. Understanding the intricate relationship between these two conditions is crucial for developing tailored therapeutic approaches that address both psoriasis and obesity concurrently.

Obesity can compromise the efficacy of various psoriasis treatments. Topical treatments, often the first line of defense, may have reduced penetration in individuals with excess adipose tissue, limiting their effectiveness. Systemic treatments, such as biologics, may also exhibit altered pharmacokinetics in obese patients, potentially requiring dose adjustments for optimal outcomes [50].

Insulin resistance, prevalent in obesity, has been linked to psoriasis development and severity [26]. It can hinder the response to certain treatments by affecting cellular processes involved in skin regeneration and immune regulation [26]. This emphasizes the need for a multifaceted approach that considers both dermatological and metabolic aspects. Obesity’s impact on inflammation and metabolism can hinder the achievement of psoriasis remission. Persistent low-grade inflammation and metabolic disturbances may undermine the effectiveness of treatments aimed at suppressing immune responses or modulating inflammatory pathways. Achieving long-term remission might require addressing obesity-related factors alongside traditional psoriasis therapies.

The treatment response in psoriasis, as well as the adverse events, can be influenced by overweight/obesity. This could be due to the nonalcoholic fatty liver disease associated with these patients and because several medications have weight-based dosing. 

### 5.1. Methotrexate

Regarding the psoriasis patients treated with MTX, obesity was found to be a greater risk factor for hepatotoxicity in comparison to alcohol, viral hepatitis, and cumulative dose of MTX, especially when other risk factors such as smoking or diabetes mellitus are present. It was suggested that liver transaminase examinations and liver biopsies should be performed more often than in non-obese psoriasis patients, at lower than 1.5 g cumulative doses [51,52]. Herron et al. reported that the efficacy of the MTX treatment of psoriasis was equal between obese and non-obese patients; however, the attrition rate of 13% in the obese group vs. 3% in the non-obese group (OR = 5.21) was explained by the lack of progress noticed in the obese patients [53]. In a similar study conducted by Murray et al., the obese psoriasis patients treated with MTX had a higher chance of losing response over time [54]. Overall, the use of MTX in obese psoriasis patients should not be discouraged, but accompanied by early liver biopsy follow-up. Because of its impact on chronic inflammation, the use of MTX is linked to a reduced likelihood of experiencing cardiovascular disease and heart attacks. This effect is particularly pronounced when the medication is administered in lower doses and in conjunction with folic acid [55].

### 5.2. Cyclosporine

Researchers investigated the connection between obesity and serum levels of cyclosporine in a group of 16 psoriasis patients [56]. They identified a robust positive correlation between the average serum concentration and the obesity index [56]. Consequently, when considering a specific cyclosporine dosage, obese individuals are more prone to exhibit elevated serum levels, heightening the risk of nephrotoxicity compared to those with normal weight. These findings remained consistent irrespective of hematocrit and plasma lipids values, implying that obesity exerts an autonomous influence on ciclosporin levels among psoriasis patients [56]. Vigilant supervision of serum drug levels, blood pressure, liver function, diabetes, and lipid profile become crucial for obese individuals undergoing ciclosporin A treatment for psoriasis. In addition to increasing the potential for toxicity among individuals undergoing cyclosporine treatment, obesity seems to impact the efficacy of this therapeutic approach. It was demonstrated that modest weight reduction (equivalent to 5–10% of body weight) resulted in enhanced treatment response when utilizing lower doses of cyclosporine in obese patients dealing with moderate to severe psoriasis [57]. Researchers studied the effectiveness of a combination of cyclosporine (2.5 mg/kg/d) with a low-calorie diet to a control group that received cyclosporine alone [57]. The study included 61 obese patients (BMI > 30 kg/m^2^) with moderate to severe psoriasis. After 24 weeks, the first group experienced an average body weight reduction of 7% (3.5%), whereas the control group saw a minimal reduction of 0.2% (0.9%). Notably, 66.7% and 86.7% of patients treated with both ciclosporin and a calorie-controlled diet achieved a 75% reduction in the Psoriasis Area and Severity Index (PASI 75) and a 50% reduction (PASI 50), respectively. This was in contrast to 29% and 48.3% of patients treated solely with ciclosporin (*p* < 0.001).

### 5.3. Acitretin

The impact of acitretin on lipid and glucose metabolism was investigated in a group of 10 psoriasis patients over a span of 1 and 3 months of treatment [58]. The alterations noted in glucose tolerance and lipid metabolism were temporary and showed no correlation with changes in BMI or the levels of TNF-α or obesity-related hormones (resistin and adiponectin) [58].

### 5.4. Biologics

A multicentric retrospective study on 504 psoriasis patients treated with biologics reported that the subjects with a BMI <30 were more likely to achieve almost complete resolution vs. the obese subjects (54.90% vs. 43.45% at week 12 and 66.84% vs. 56.55% at week 24) [57]. Related to BMI, the long-term efficacy of secukinumab and ustekinumab recorded a drop (associated with a higher attrition rate), while adalimumab, etanercept, and ixekizumab were not negatively influenced [57]. 

In the study by Higa-Sansone et al. [58], a case was documented wherein psoriasis achieved full remission due to weight loss achieved through bariatric surgery. The authors suggested that bariatric surgery could be considered as a viable treatment choice for psoriasis among individuals who are severely obese.

A multicentric retrospective study on 504 psoriasis patients treated with biologics reported that the subjects with a BMI <30 were more likely to achieve almost complete resolution vs. the obese subjects (54.90% vs. 43.45% at week 12 and 66.84% vs. 56.55% at week 24) [59]. Related to BMI, the long-term efficacy of secukinumab and ustekinumab recorded a drop (associated with a higher attrition rate), while adalimumab, etanercept, and ixekizumab were not negatively influenced [59]. 

In the study by Higa-Sansone et al. [60], a case was documented wherein psoriasis achieved full remission due to weight loss achieved through bariatric surgery. The authors suggested that bariatric surgery could be considered as a viable treatment choice for psoriasis among individuals who are severely obese.

Naldi et al. [61] demonstrated an inverse correlation between the consumption of fresh vegetables and fruits and the severity of psoriasis. However, it is important to note that this study did not take the caloric intake into consideration.

In conclusion, the impact of obesity on psoriasis treatment is a multidimensional challenge that underscores the need for integrated care. Recognizing the intricate interplay between these conditions and considering their combined effects can lead to more effective and individualized treatment strategies, ultimately improving the quality of life for individuals living with psoriasis and obesity. Managing psoriasis and obesity requires a holistic approach that addresses both conditions simultaneously. Lifestyle modifications, including weight loss, exercise, and a balanced diet, can help reduce inflammation and improve psoriasis symptoms.

### 5.5. Semaglutide

Semaglutide is an authorized therapeutic agent for the management of type 2 diabetes mellitus that functions as a prolonged-acting agonist of the glucagon-like peptide 1 (GLP-1) receptor [62]. Semaglutide is also one of the most effective available treatments against obesity, due to increased fullness sensation and decreased appetite [63]. GLP-1 agonists improved clinical psoriasis in a small number of patients with type 2 diabetes mellitus and obesity, associated with a decrease in IL-17 and γδ T-cell number [64,65].

### 5.6. Other Hypolipedemic Drugs

Statins can be used for the treatment of hyperlipidemia and have been proven useful in psoriasis for reducing the severity, lowering the cardiovascular risk, and improving the efficacy of topical corticosteroids [66,67,68]. Statins are also associated with a decreased overall risk of psoriasis [69]. The beneficial effects of statins on psoriasis can be related to selective inhibition of leukocyte function antigen-1, inhibition of leukocyte–endothelial adhesion, stimulation of T natural killer cells, and lowering of C-reactive protein, TNF-α, IL-1, and IL-6 [66,70]. In some studies, statins had a neutral or even detrimental effect on psoriasis; therefore, a personalized approach is indicated when considering this drug [71].

The effect of fibrates and glitazones on the treatment of psoriasis is controversial, with less supporting evidence, providing a path for further studies [71].

## 6. The Microbiome in Psoriasis

The microbiome and its connection to psoriasis constitutes a growing topic of medical research. The microbiome represents the set of microorganisms (bacteria, viruses, fungi) that live inside and on the surface of the human body, having a significant impact on health. In patients with psoriasis, a possible link between microbiome imbalances and the onset or worsening of the condition has been observed.

Recent studies have revealed differences in the composition of the skin microbiome of psoriasis patients compared to healthy controls. Certain bacterial species may be more or less abundant in these patients. Microbial imbalances can influence the immune system and inflammation, thus playing a role in the development and progression of psoriasis.

The human microbiome, which consists of the entire microorganisms that reside inside and outside the human body, is influenced by nutrition and obesity [72]. Whole-bacterium translocation [73,74], accumulation of bacterial metabolites (short-chain fatty acids) [75,76], and altered tryptophan metabolism [77,78] are some mechanisms which link microbiome imbalances to autoimmune conditions. Infections with group A β-hemolytic streptococci [79], *Staphylococcus aureus*, and fungi [80] are well-known triggers and exacerbating factors for psoriasis. However, the influence of the microbiome in psoriasis is not so well understood. IL-17 production is influenced by the gut microbiome and could partially sustain the pathogenesis of psoriasis [22,81]. Th17 cells and the cytokines they produce play a significant role in the advancement of psoriasis and the underlying mechanisms of obesity. This suggests that probiotics, known for their efficacy in addressing obesity, could also potentially offer a viable approach for treating psoriasis. Microbiomic data could provide precise molecular signatures for the diagnosis of psoriasis through deoxyribonucleic acid (DNA) sequencing and downstream analysis protocols [82]. Specifically, in psoriasis, there is an overrepresentation of Escherichia coli [83], while the Coprococcus genus is underrepresented [84].

The gut microbiome of obese individuals exhibits lower diversity compared to non-obese individuals [85,86]. This reduced diversity is marked by a decrease in Gram-negative bacteria, particularly those belonging to the Bacteroidetes group, and an elevation in Gram-positive Firmicutes bacteria, which can also be found in psoriasis patients [85,86].

A summary of the skin and gut microbiome in psoriasis patients is represented in Table 2 and Table 3 [87].

Probiotics, prebiotics, and synbiotics have been studied in psoriasis, in animal and human models, with promising results involving Lactobacillus pentosus GMNL-77, Lactobacillus sakei proBio-65, Lactobacillus sporogenes, and Bifidobacterium infantis 35624 [88].

Lactobacillus casei strain Shirota (LAB13) and Bifidobacterium infantis have been confirmed to reduce obesity not only in animals, but also in humans [88]. Lactobacillus probiotics have been successfully used in vitro in imiquimoid-induced psoriasis in mice, resulting in lower gene expression levels of pro-inflammatory markers such as TNF-α, IL-19, IL-17A, and IL-23, as well as lower PASI scores [89,90]. A 6–8-week oral administration of Bifidobacteria infantis 35624 in psoriasis patients not receiving any anti-psoriatic treatment significantly decreased the plasma levels of C-reactive protein and TNF-α [91].

However, research is still in its early stages, and the complexity of interactions between the microbiome and psoriasis requires further investigation. Therefore, the connection between the microbiome and psoriasis is a topic of major interest in medicine, opening new perspectives in understanding and addressing this condition. Research in this area could lead to the development of innovative therapeutic strategies, thus improving the lives of people affected by psoriasis. In the future, personalized treatments that take into account the individual microbiome could represent a promising direction in the management of psoriasis. There is a lack of conclusive evidence regarding the long-term impacts of probiotics, whether utilized as dietary supplements or as supplementary therapeutic measures. As a result, it is imperative to thoroughly evaluate the safety of these bacterial agents before considering their use in the management of diverse medical conditions.

## 7. Recommendations for the Obese Psoriasis Patient

- Patient education on the link between psoriasis and obesity;
- Dietary counseling;
- Lifestyle counseling to combat obesity;
- Psychological and/or psychiatric support to prevent or treat psychological stress and other mental health disorders;
- Consideration of the use of probiotics in specific patients;
- Consideration of the use of hypolipidemic drugs in specific patients;
- Consideration of bariatric surgery in specific patients.

## 8. Discussion

In conclusion, there is a clear association between obesity and psoriasis. It is reasonable to argue that obesity can increase the risk of developing psoriasis and vice versa. The pathophysiology of psoriasis and obesity is intertwined through shared inflammatory pathways and immune dysregulation. Understanding these complex interactions is essential for providing comprehensive care to individuals affected by both conditions. A multidisciplinary approach that addresses the underlying mechanisms of both psoriasis and obesity is key to improving patient outcomes and quality of life.

Systemic treatment of psoriasis has several challenges, such as decreased compliance, drug toxicity, and lack of prolonged clinical remission. Effort should be put into addressing modifiable factors influencing the course of psoriasis, including obesity. Future clinical studies should assess the efficiency of special diet regimens, such as the Mediterranean diet and pre- and probiotics in obese patients suffering from psoriasis.

## Figures and Tables

**Table 1:** Pathophysiology of psoriasis and obesity.

Pathophysiology of Psoriasis and Obesity	Mechanism
Inflammation	Obesity triggers low-grade chronic inflammation, which can exacerbate psoriasis by promoting cytokine release and immune system dysregulation [20].
Insulin resistance	Insulin resistance, common in obesity, may contribute to psoriasis by affecting keratinocyte proliferation and inflammation [21].
Adipokines	Adipose tissue secretes adipokines, which can influence immune responses and skin inflammation in psoriasis [17].
Gut microbiota	Obesity-related changes in gut microbiota composition can impact systemic inflammation and exacerbate psoriasis through microbiota–skin axis interactions [22].
Genetic factors	Shared genetic susceptibility between psoriasis and obesity may contribute to their co-occurrence [23].

**Table 2:** The skin microbiome in psoriasis [87].

Increased Skin Bacteria in Psoriasis	Decreased Skin Bacteria in Psoriasis
Firmicutes	Actinobacteria
Streptococci	Staphylococci
Proteobacteria	Propionibacterium
Corynebacterium	Acidobacteria Gp4
	Schlegelella
Staphylococci	Firmicutes
Rhodobacteraceae	Anaerococcus
Campylobacteraceae	Deinococcus
Moraxellaceae	
Prevotella	
Brevundimonas	
Finegoldia	
Neisseriaceae	
Micrococcus	

**Table 3:** The gut microbiome in psoriasis [87].

Increased Gut Bacteria in Psoriasis	Decreased Gut Bacteria in Psoriasis
Enterococcaceae	Akkermansia
Clostridium citroniae	
Streptococcus	Coprococcus
Bifidobocterium	Coprobacillus
Akkermansia	Verrucomicrobia
Faecalibacterium	Jenericutes
Firmicutes	Mollicutes
Ruminococcus	Bacteroides
Megasphaera	Lachnospira
Blautia	Collinsella
Dorea	Sutterella
Christensenella	
Parabacteroides	
Lactococcus	

## Data Availability

No new data were created or analyzed in this study. Data sharing is not applicable to this article.
